# Health technology assessment infrastructure for emirates health services: a phased framework

**DOI:** 10.3389/frhs.2025.1662561

**Published:** 2025-10-23

**Authors:** Suad Hannawi, Rita Ojeil, Abdalla Alnaqbi, Khadija Almarshoodi, Essam Alzaaroni, Abdulaziz Alzaaroni, Noor Almoheiri, Sondos Al-Salmi, Haifa Hannawi, Maria Al-Salmi, Sabaa Saleh Al-Hemyari

**Affiliations:** ^1^ Al Kuwait Hospital Dubai, Emirates Health Services (EHS), Dubai, United Arab Emirates; ^2^ Market Access and HEOR, Carexso, Dubai, United Arab Emirates; ^3^ Clinical Services Sector, Emirates Health Services (EHS), Dubai, United Arab Emirates; ^4^ Financial and Support Services Sector, Emirates Health Services (EHS), Dubai, United Arab Emirates; ^5^ Mohammed Bin Rashid University of Medicine and Health Sciences, Dubai, United Arab Emirates; ^6^ The College of Health Sciences, University of Sharjah, Sharjah, United Arab Emirates

**Keywords:** health technology assessment, impact analysis, cost-effectiveness, managed entry agreements, multi-criteria decision analysis, value-based healthcare, United Arab Emirates

## Abstract

This policy brief presents a structured, phased framework to establish Health Technology Assessment (HTA) within Emirates Health Services (EHS), promoting evidence-based decision-making and optimizing healthcare delivery. The model emphasizes capacity building, stakeholder engagement, and transparency to enhance patient outcomes and ensure financial sustainability. It follows four phases: Phase 0 (strategy alignment, stakeholder mapping), Phase 1 (budget impact and disease burden analysis), Phase 2 (managed entry agreements, multi-criteria decision analysis), and Phase 3 (full HTA adoption with performance metrics). Key methodologies include budget impact modeling, cost-effectiveness, and cost-utility analysis. Evidence is drawn from registries, clinical databases, and pharmacoeconomic studies. The framework offers a scalable and sustainable roadmap for HTA in the UAE, supporting efficient technology integration, optimal resource allocation, and alignment with national priorities. However, the framework's successful implementation will depend on the availability of high-quality local data, sustained stakeholder engagement, and supportive regulatory mechanisms. Potential challenges such as resource constraints, fragmented governance, and variability in HTA maturity across the region highlight the need for flexibility and adaptive strategies.

## 1 Introduction

The healthcare industry is shifting from “volume to value”. This transformation is evident in the insurance sector as well, through the emergence of alternative payment models, and the provider sector, with new organizational structures (1). Value in health care refers to improving health outcomes at lower costs, aiming to enhance patient care through improved care delivery and value-based transformations (2, 3).

The rise of innovative technologies has significantly improved health indicators. However, they have substantial financial implications, and the impact and value of some innovations may not be fully documented. Health technology assessment (HTA) is a globally recognized process for evaluating the incremental value of such innovations (4).

**Figure 1:**
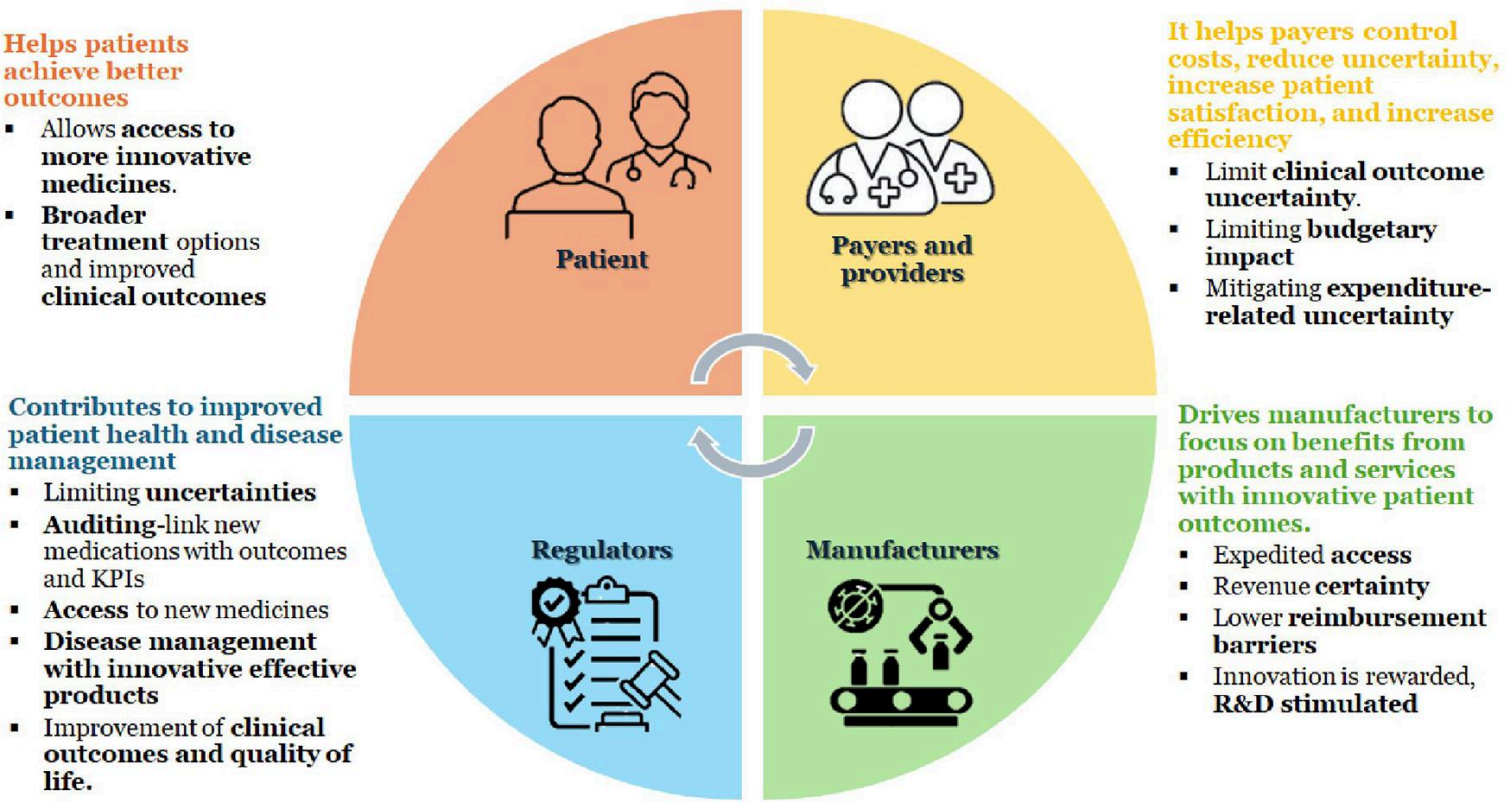
Benefits of HTA. KPI, key performance indicators; R&D, research and development.

HTA interventions aim to improve health, treatment, support rehabilitation, or improve the organization of health care delivery (5). It is an essential activity to ensure efficient use of resources and enhance coverage decisions (6, 7). HTA aims to improve healthcare treatment by helping stakeholders make informed, efficient, and cost-effective healthcare decisions, maximizing resource use. It bridges research and policy, guiding decisions on reimbursement and implementing new health technologies within a national healthcare system (8). It evaluates various aspects affected by the introduction of a new health technology, categorized into four key areas: the technology itself, the patient, the organization, and the economy (7). Figure 1 illustrates the overall benefits of HTA. It is known that HTA supports decision-making, reimbursement, and universal coverage, yet one-third of countries in the world lack a formal HTA process (4).

While HTA is globally acknowledged, its institutionalization in the UAE remains nascent. As of 2016, there was no formal HTA body, Emirate-level health authorities handled key decisions. In 2018, the Department of Health-Abu Dhabi launched an HTA unit and even published dedicated HTA guidelines in mid-2025 to guide the evaluation of high-cost therapies and devices (9). Beyond Abu Dhabi boundaries, HTA adoption remains limited. Meanwhile, regional momentum includes Saudi Arabia's recently mandated HTA for high-cost drugs through the high-cost medication committee (10), Tunisia's central HTA agency Instance Nationale d'Evaluation et d'Accreditation en Sante (INEAS), and Jordan's hospital-level HTA at the King Hussein Cancer Center (10). These examples chart a broader shift in GCC and MENA toward structured, transparent HTA.

The UAE is among the leading countries globally in adopting innovative health technologies, underscoring the importance of exploring key elements needed to establish an HTA framework (4). Given the current fragmentation of the global HTA landscape, there is a pressing need to develop standardized HTA frameworks. Global variation in HTA structures, methods, and outputs underscores this fragmentation and the parallel push for standardization. Comparative analyses, with key good practice elements such as deliberative processes and governance, are still inconsistently defined or implemented (11). Regionally, MENA comparisons likewise highlight heterogeneous maturity and practice, reinforcing the need for context-sensitive, yet standardized, frameworks (10). In addition, HTA institutionalization by forming legislation towards it is also needed. Once this legislation is made, its integration into decision-making can be formally realized (12). Considering this, the Emirates Health Services (EHS) targets to establish a dedicated health technology assessment infrastructure.

In this paper, we suggest a phased HTA infrastructure and its anticipated outcomes post-implementation. This will help in supporting decision-making with evidence-based recommendations on healthcare technology effectiveness, safety, and cost. It will also assess the financial and health outcomes of new healthcare technologies, guiding decision-making, reimbursement, and efficient resource allocation.

## 2 Methods

**Figure 2:**
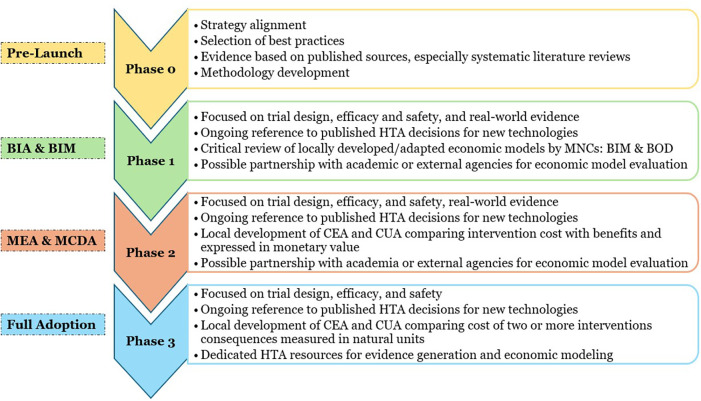
Framework of the phased approach to HTA implementation. BIA, budget impact analysis; BIM, budget impact modeling; BOD, burden of disease; HTA, health technology assessment; MEA, managed entry analysis; MCDA, multi-criteria decision analysis; CEA, cost-effectiveness analysis; CUA, cost-utility analysis.

Building an effective HTA infrastructure is a complex process requiring careful planning and staged execution. To achieve these objectives, the EHS proposes a staggered, phased approach aligned with the global HTA standards. Figure 2 gives the framework of the 4 phased approach to HTA implementation.

### 2.1 Phase 0: pre-launch activities in building a robust HTA infrastructure (period: 0–3 months)

This phase focuses on pre-launch health economic activities aimed at aligning strategies, synthesizing evidence, and identifying and incorporating best practices and methodologies. This is the most crucial phase, as it lays the foundation for the subsequent stages, ensuring that all key stakeholders and resources are adequately prepared to implement HTA in a structured and effective manner. This phase includes strategy alignment that involves the selection of core team members from the national health regulatory authority, training stakeholders, aligning methods and model types, and developing a communication plan. This would be followed by selecting the best practices focusing on technology selection, contracts, data access, transparency and evidence synthesis involving data collection, analysis, and tools like multi-criteria decision analysis (MCDA) and disease registries. The last step of this phase is methodology development, which covers model creation, expert selection, stakeholder profiling, and timeline setting.

In the UAE context, this phase must account for fragmented HTA activities, currently defined only in Abu Dhabi via formal guidelines released in 2025 (9). Replicating this structure nationally will require coordination across EHS and alignment with regional precedents such as Saudi Arabia's HTA policy for high-cost medication.

The proposed timelines for each phase are informed by international HTA adoption patterns and local system readiness. A 0–3-month window for Phase 0 is primarily preparatory, focusing on stakeholder mapping, training, and evidence synthesis, which can be completed in a short, intensive cycle. Early international experiences, such as Thailand and Saudi Arabia, show that pre-launch strategy alignment can be accomplished rapidly when supported by strong regulatory leadership (6, 7).

### 2.2 Phase 1: budget impact analysis (period: 3–24 months)

This phase focuses on the creation of a budget impact analysis (BIA) to assess the financial sustainability of new healthcare technologies and the burden of disease (BOD) that helps in understanding the economic, clinical, and humanistic burden of diseases, which can vary significantly across regions due to factors like ethnicity, environmental exposure, and socioeconomic conditions (13). This phase is integral to building a cost-conscious healthcare system that prioritizes value for money and effective patient outcomes.

#### 2.2.1 Budget impact modeling

Budget impact modeling (BIM) involves the collection of relevant data and documents, including clinical data (efficacy and safety), pricing information, patient demographics, and existing healthcare resource utilization. During the kick-off, key objectives and timelines for BIM development are set. It also ensures alignment among stakeholders on methodology, scope, and data collection processes. This is followed by a literature review and secondary research to gather evidence on disease, treatment, and costs; identify data gaps, compare current vs. new technology costs, and define economic assumptions for BIM development. Once sufficient data has been collected, the next step is model concept development, which involves defining the structure of BIM. Data gaps are addressed through a discussion guide, used in expert interviews to refine the model for local healthcare needs. In BIM development, the model is built or adjusted using validated data to simulate budgetary impact. The results and reporting stage involves the generation of a report on budget implications to ensure model accuracy through quality checks followed by a presentation of the findings to decision-makers.

#### 2.2.2 Burden of disease analysis

It includes measures like disability adjusted life years (DALYs), which represent the sum of years of life lost (YLL) due to premature death and years lived with disability (YLD). Accurate estimation of the BOD provides essential information for healthcare policymakers and stakeholders, enabling them to allocate resources effectively, prioritize interventions, and improve disease management. Ultimately, this contributes to better patient outcomes and enhances public health initiatives.

The BOD analysis starts with a material review and an initial meeting to define the scope. This is followed by the collection of epidemiological and economic data through a literature review, and local insights through expert interviews. A model is then developed to assess costs, including DALYs, YLL, and YLD, which would be adapted to the UAE, considering complications and productivity losses. The results will highlight the disease burden and the need for better treatments.

The 3–24-month duration for Phase 1 is justified by the resource-intensive nature of BIM and BOD assessments. These analyses require robust epidemiological data, literature reviews, expert validation, and model testing, which realistically demand extended timelines (6, 7). BIM will primarily use cost-minimization analysis (CMA) when equivalent clinical outcomes are demonstrated, and budget impact modelling frameworks aligned with International Society for Pharmacoeconomics and Outcomes Research (ISPOR) guidelines. BOD studies will rely on DALYs and productivity loss calculations, ensuring both economic and humanistic perspectives are captured (5, 7).

### 2.3 Phase 2: MEA and MCDA (24–36 months)

This phase is dedicated to the implementation of managed entry agreements (MEAs) and MCDA. The primary goal is to develop innovative contract systems and frameworks to evaluate and manage costly, innovative healthcare technologies and drugs, particularly those with incomplete data or high uncertainty. This phase focuses on ensuring that new, innovative, and costly healthcare technologies can be introduced in a financially responsible and evidence-based manner.

The main objectives of phase 2 are ensuring financial sustainability, improving access, and providing robust evidence for informed decision-making.

A 24–36-month period is proposed for Phase 2, as MEAs and MCDA frameworks typically require multi-stakeholder negotiation, real-world data collection, and the testing of innovative contracting mechanisms (13). Economic evaluations in this phase will include cost-effectiveness analysis (CEA), expressed in incremental cost-effectiveness ratios (ICERs), and cost-utility analysis (CUA) using QALYs as an outcome measure. These methods provide decision-makers with robust value-for-money assessments while accommodating uncertainty around novel technologies (5, 13).

### 2.4 Phase 3: full adoption of HTA with the establishment of key performance indicators

In Phase 3 the attention is diverted towards the establishment of a sustainable HTA framework by defining roles, responsibilities, and key performance indicators (KPIs) for an effective integration into the healthcare system. Key components include stakeholder and model selection with KPIs covering evaluation timeliness, budget impact, evidence quality, and stakeholder satisfaction.

In this phase, HTA practices become fully integrated into the healthcare system. The use of HTA in decision-making involves the adoption of tools such as BOD analysis and humanistic outcomes including patient outcomes and quality of life. Strategic communication plays a critical role in fostering collaboration among various entities, including regulatory authorities, healthcare providers, multinational companies, and patients. It adopts a strategic approach that aligns all stakeholders toward a common vision for HTA implementation.

Phase 3 is expected to take 36–60 months to achieve full integration, as institutionalization of HTA requires legislative adoption, capacity building, and iterative refinement of processes (6, 12). Key economic models in this stage will expand beyond BIM and CEA to include Cost-benefit analysis (CBA) for broader policy trade-offs, and scenario analyses to anticipate long-term system-level impacts. The use of mixed-methos economic models ensures that both quantitative and qualitative aspects of health technology adoption are systematically addressed.

This four-phase approach is designed to sequentially build EHS's HTA capacity, from initial strategy alignment to full institutionalization. Each phase's outputs inform the next phase's activities, ensuring continuity. The stepwise progression from one phase to another aligns with global best practices for HTA implementation, where clear roadmaps and short vs. long-term action plans provide decision-makers with a structured pathway. The results of each phase can be directly traced back to its corresponding methodology, developing a coherent narrative from initial planning to evidence-based outcomes.

## 3 Results

The results of implementing this phased HTA framework are organized in alignment with the four phases described above. The proposed framework highlights the value of cost-effectiveness, patient-centered outcomes, and stakeholder collaboration. It ensures the prioritization of patient well-being in conjunction with cost efficiency. The establishment of HTA as a systematic, transparent, and evidence-based decision-making process will have a lasting impact on the quality, efficiency, and sustainability of healthcare. Ultimately, this will ensure that patients receive the most effective and value-driven care available.

### 3.1 Expected outcomes

**Figure 3:**
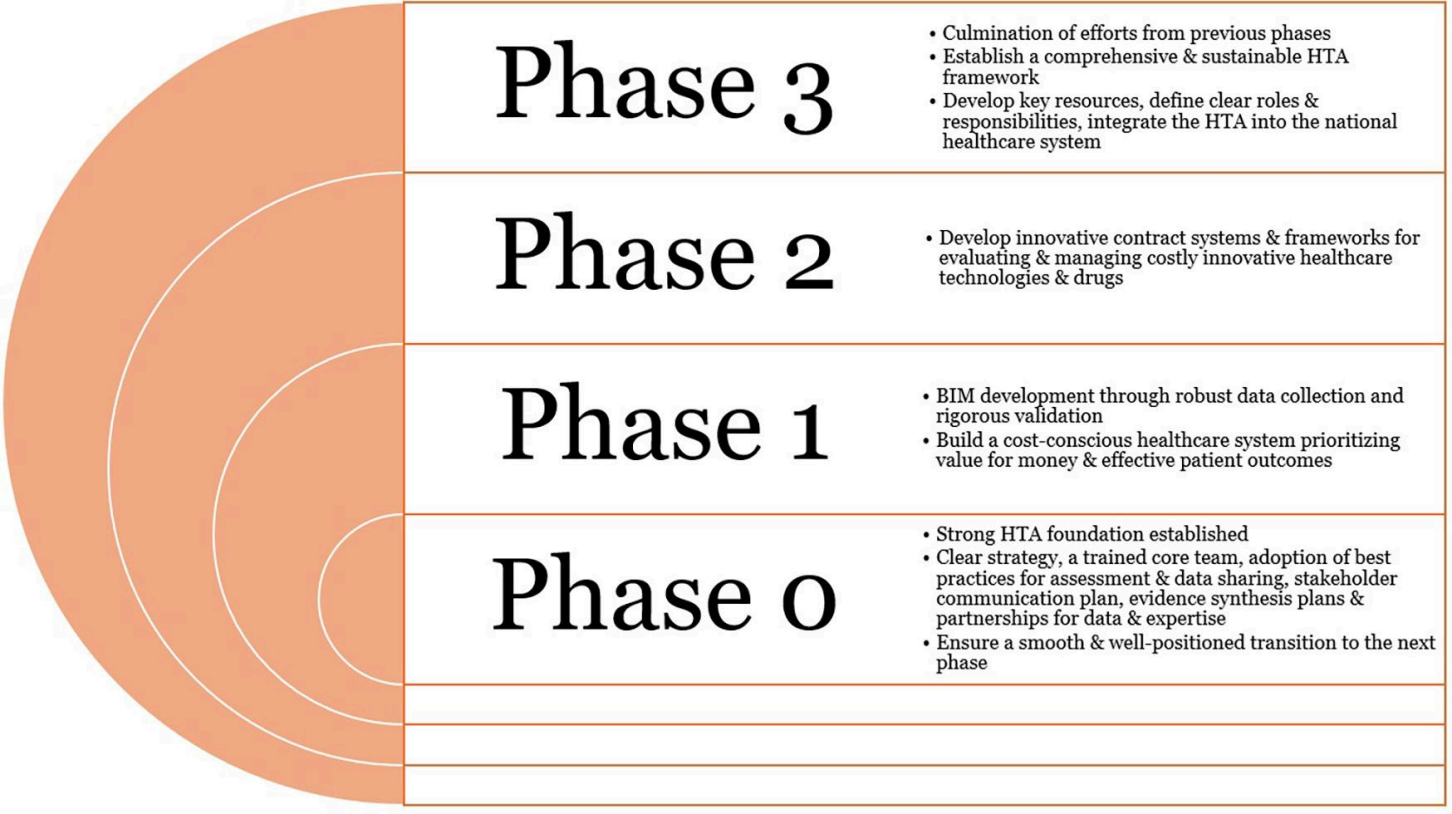
Expected outcomes from the phased approach to the HTA implementation. HTA, health technology assessment.

Figure 3 depicts the expected outcomes from the different phases of HTA implementation. Phase 0 produces a foundation of governance and data resources that all subsequent phases will build upon, mirroring the approach of successful international HTA roadmaps that emphasize early stakeholder engagement and capacity building. In Phase 1, BIM and BOD yield crucial data on financial sustainability and population health needs, enabling evidence-based prioritization of technologies. The outcome in Phase 2 is an innovative contracting system and decision criteria matrix that allows the introduction of high-cost technologies with controlled risk. This phase ensures that real-world evidence and cost-effectiveness data directly inform coverage decisions, as recommended by global HTA experts. By the end of Phase 3, HTA processes are institutionalized and routinely integrated into healthcare decision-making. Key performance indicators such as assessment turnaround time, budget impact accuracy, and stakeholder satisfaction are tracked to measure success and drive continuous improvement.

### 3.2 Strategic approaches to HTA development: building capacity, engagement & transparency

Capacity building involves the development of high-quality local data sources, such as disease and patient registries, pharmacoeconomic studies, systematic literature reviews, and other publications to enhance country-specific data availability. Collaboration with academia, key opinion leaders, multinational corporations, and external agencies is essential for generating comprehensive databases. Identifying and establishing dedicated HTA resources for evidence generation and economic modelling is key to supporting decision-making processes. These efforts ensure a foundation for effective evaluation of health technologies, combining clinical and economic insights to inform healthcare strategies and policies. In the short to mid-term, capacity building can be achieved by establishing an HTA committee under EHS in collaboration with a third-party organization.

A well-defined communication plan is critical for the successful implementation of an HTA framework to ensure that all stakeholders are engaged, informed, and aligned with the goals and activities of the HTA process. The communication plan focuses on stakeholder engagement as well as internal and external communication.

HTA stakeholders include government bodies, healthcare providers, manufacturers, patients, and payers. Clear communication ensures transparency through regular updates**,** public consultations and partnerships with manufacturers to align evidence requirements.

To maximize policy relevance, stakeholder roles should be concretely embedded within the HTA process phases. For instance, patient representatives can be systematically engaged during scoping (Phase 0) to ensure outcomes reflect lived experiences, while payers and insurers can guide BIA assumptions in Phase 1. Clinicians and academic experts play critical roles in validating MCDA criteria in Phase 2, whereas policymakers are central to setting key performance indicators and monitoring institutionalization in Phase 3. Experiences from the National Institute for Health and Care Excellence (NICE) and Canadian Agency for Drugs and Technologies in Health (CADTH) demonstrate that codifying stakeholder responsibilities not only enhances transparency but also strengthens legitimacy and public trust in HTA decisions (11, 14, 15).

Effective internal communication within the HTA governance ensures seamless decision-making and operational efficiency. Clear coordination among the governing body, working groups, and experts to align priorities and methodologies, enhancing assessments and timely decisions.

Transparent engagement with the healthcare community, policymakers, healthcare providers, and the public builds trust. Strategies include publishing HTA reports, developing policy briefs, and providing educational materials to healthcare providers.

### 3.3 Enhancing evidence through peer review and interim analyses

Peer-reviewed publications and interim analyses enhance evidence quality and accessibility, providing reliable data for policymaking. These resources strengthen HTA infrastructure and support evidence-based decisions.

Publishing findings in peer-reviewed journals ensures data quality and transparency, building trust among healthcare stakeholders. Real-world evidence is generated through interim analyses from health economic evaluations and disease registries, providing timely insights into healthcare interventions' effectiveness, cost-efficiency, and patient outcomes. Aligning evidence generation with global HTA standards, such as BIM, cost-effectiveness analysis, and cost-utility analysis, ensures the UAE's healthcare system remains competitive and credible.

Effective evidence used in decision-making ensures informed and equitable healthcare policies. High-quality, peer-reviewed data and real-world evidence provide critical insights into the clinical, economic, and patient-centered outcomes of healthcare interventions that support policy formulation, resource allocation, and the implementation of MEAs, linking reimbursement to real-world performance. Challenges like limited infrastructure, data inconsistencies, and insufficient training among stakeholders can be addressed by a centralized HTA body, transparent evidence dissemination, and setting clear key performance indicators for evidence quality and expanded training programs.

### 3.4 The proposed organizational framework for a transparent HTA process within EHS

**Figure 4:**
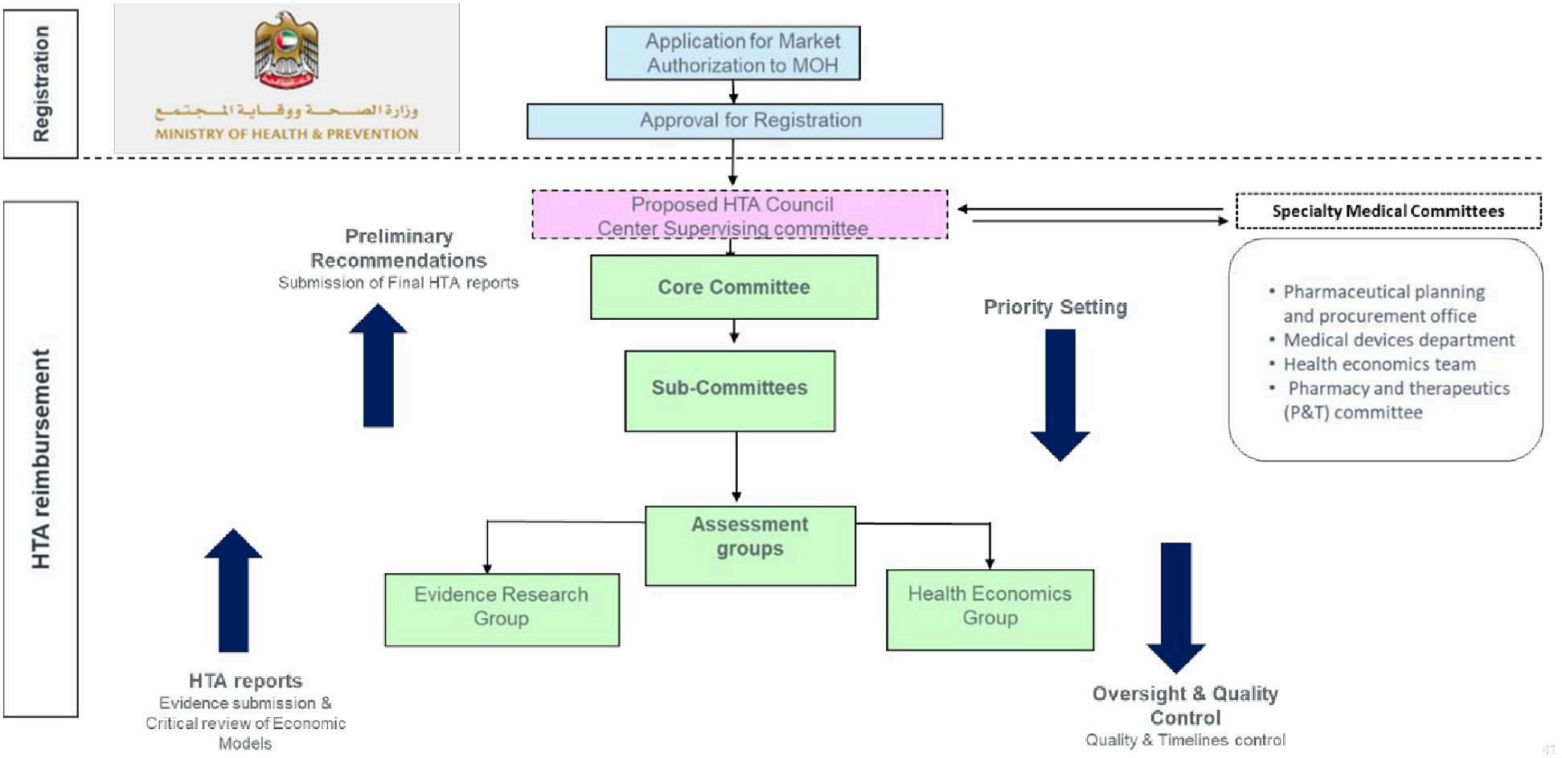
Proposed organizational framework for HTA implementation.

The HTA framework involves a core committee and sub-committees supported by evidence research and health economics groups. Specialty medical committees provide input on setting priorities, while oversight and quality control ensure quality and adherence to timelines. Figure 4 shows the proposed organizational framework.

The guiding principles include a national HTA remit, using economic evaluation through patient, intervention, comparison, outcome (PICO) framework, and stakeholder engagement. HTA activities cover medicine, devices, and diagnostics, with prioritization based on disease burden, economic impact, and public interest. The proposed operational framework in the UAE involves a transition from BOD to BIA and also includes MEA and MCDA. The HTA body will be patient-centric, autonomous, and transparent, ensuring public availability of recommendations.

## 4 Discussion

Collectively, these phased outcomes demonstrate how a structured HTA implementation can transform evidence into policy. Results of each phase build on the previous one, creating a continuous feedback loop from analysis to decision-making. This integrated progression addresses the initially identified challenges, including fragmentation, data gaps, etc., by ensuring that methods, results, and policy decisions are tightly connected.

This phased approach creates a clear path from methods to outcomes, which is key for stakeholder support and policy adoption. Building a robust HTA framework in the UAE requires learning from international experiences while tailoring the approach to local realities. Globally, HTA implementation shows both common patterns and stark variations. Some countries have adopted HTA into their health systems through independent national agencies, whereas others use it sparingly or rely on decentralized models (16). The United Kingdom and Canada established formal HTA agencies decades ago to guide reimbursement decisions, applying explicit cost-effectiveness criteria. In contrast, the United States has historically lacked a single national HTA body, leading to more fragmented, payer-specific assessments (16). Even among countries that conduct economic evaluations as part of HTA, there are differences in emphasis; some stress QALYs as the measure of value, while others employ alternative notions of “value” beyond QALYs. Underlying these differences are each nation's unique institutional context, social values, and health system priorities; accordingly, experts note that there is no one-size-fits-all HTA model applicable worldwide. Successful HTA programs must align with a country's decision-making culture and healthcare structure, a principle that reinforces the UAE's need to craft its own context-sensitive roadmap.

Experiences from other countries, both regionally and globally, highlight practical strategies and challenges that resonate with the UAE's situation. Many nations, especially in emerging healthcare systems, have adopted phased or focused approaches when introducing HTA. For instance, Thailand rapidly institutionalized HTA through the establishment of its Health Intervention and Technology Assessment Program (HITAP) in the 2000s, which allowed cost-effectiveness evidence to directly inform the expansion of its universal health coverage benefits (6). This demonstrated how early government support and capacity-building can integrate HTA into national decision-making. In Tanzania, a more resource-constrained setting, HTA methods were initially applied to update the national essential medicines list; from 2014 to 2018, the country formed an official HTA committee that successfully used evidence-based criteria to revise its formulary (17). This stepwise integration of HTA into an existing prioritization process shows that even without a standalone agency, HTA can be institutionalized through focused initiatives that build experience and trust in the process.

The transformation of healthcare from a “volume to a value-based approach” has been the foundation stone for the development of HTA. UAE is proactively upgrading its healthcare framework by adopting innovative healthcare technologies in conjunction with ensuring improved patient outcomes and cost-effectiveness of healthcare services. Experiences from other countries, both regionally and globally, highlight practical strategies and challenges that resonate with the UAE's situation. Many nations, especially in emerging healthcare systems, have adopted phased or focused approaches when introducing HTA. As seen in Thailand and Tanzania, it has been demonstrated how early government support and capacity-building can integrate HTA into national decision-making. This comprehensive assessment of healthcare technologies, which includes medical devices, pharmaceuticals and interventions, ensures that healthcare systems prioritize value-driven investments. The emphasis of the UAE on HTA perfectly aligns with the global trend, thereby recognizing the importance of structured frameworks in enhancing patient care, reducing economic burden and overall improvement of health quality.

Despite the potential advantages of HTA, certain challenges are obstructing its seamless integration into healthcare decision-making. These include limitations of resources, lack of standardized guidelines, poor digital infrastructure, insufficient local data, and cultural barriers. Addressing these challenges requires strategic investments in stakeholder engagement, the development of high-quality data, capacity building and a regulatory framework that supports the implementation of HTA. The phased approach outlined here provides a structured roadmap for overcoming these hindrances and ensuring a well-developed HTA system.

Concrete mechanisms are emerging to bridge these gaps. In Abu Dhabi, the Department of Health has co-developed a roadmap for HTA institutionalization using evidence-informed deliberative processes, highlighting the value of multi-stakeholder engagement and structured governance in overcoming fragmentation and data limitations (4, 10, 18). To strengthen local capacity, HTA experts from the region recommend expanding graduate and postgraduate training programs, underpinned by academic-public partnerships, to ensure sustainable skill development and align curricula with international best practices (10). The UAE can leverage these models to provide both technical and policy-focused education in health economics, outcomes research, and decision sciences.

Within the MENA region, HTA initiatives are gaining momentum, though each country has approached it differently. Saudi Arabia has taken a top-down initial step by mandating HTA reviews for high-cost medicines. A high-Cost Medication Committee under the Saudi Health Council now requires evidence of value for expensive new drugs, and a dedicated national HTA center is being established under the Ministry of Health to support this process (10). The Saudi experience illustrates a common starting point for HTA in the region, focusing on high-budget impact technologies, coupled with plans to expand once capacity matures. Neighboring countries have adopted other models; Tunisia has arguably the most institutionalized HTA in the Arab world, via INEAS. It is a government-backed body under the Ministry of Health, evaluating new medicines and health technologies with an emphasis on those that are high-cost or have a significant population impact. Notably, Tunisia places a strong emphasis on transparency and local capacity. INEAS publishes its HTA reports and recommendations on its website for public access, and it has invested in training both its staff and other stakeholders, including policymakers and clinicians in HTA concepts. Meanwhile, Jordan represents a more limited but instructive use case, rather than a national program, Jordan's flagship cancer hospital (King Hussein Cancer Center) established an HTA unit to assess expensive oncology drugs before formulary adoption. This hospital-level HTA model addresses urgent needs in a specialized context and demonstrates how, in the absence of a nationwide system, individual institutions can still implement HTA principles to improve decision-making. The varied MENA experiences underscore that while the impetus for HTA is widespread, driven by rising costs and the value agenda, the mechanisms of implementation differ.

In the UAE, HTA activity continues to be sporadic, primarily Abu Dhabi-based. This fragmented landscape underscores the urgency for a centralized legal framework and capacity building across all emirates. Regionally, expansion in Saudi Arabia, Tunisia, and Jordan illustrates both promising models and adoption challenges (9). Together, these reinforce the need for a phased and scalable HTA framework tailored to the UAE's context.

To address uneven adoption across emirates, a pilot technology-driven HTA system is underway in Dubai (2025–27), featuring a mixed-methods evaluation that includes electronic surveys, stakeholder interviews, and digital infrastructure mapping, to inform the development of a scalable national application (19). Such initiatives can serve as proof-of-concept for digital governance, data integration, and streamlined stakeholder alignment.

Moreover, the UAE is advancing economic evaluation capabilities; recent research has established cost-effectiveness thresholds (CETs) tailored to the UAE context, offering decision-makers quantifiable benchmarks that reflect both international standards and domestic healthcare valuations (20).

## 5 Conclusion

This policy framework establishes HTA infrastructure within the EHS using BIA, disease registries, and MEA for informed decision-making and financial sustainability of innovative healthcare technologies. The phased implementation and focus on stakeholder engagement, transparency, and patient-centered care are key to embedding HTA into the national healthcare system.

The framework emphasizes a comprehensive, evidence-based approach to improve access, efficiency, and patient outcomes while enhancing transparency and accountability. Fostering collaboration between policymakers, healthcare providers, payers, and manufacturers ensures the alignment of health priorities with resource allocation. The UAE's HTA development journey thus mirrors a broader GCC/MENA transformation towards evidence-based, value-driven healthcare decision-making, offering opportunities for cross-country learning and collaboration.

For policymakers, this means prioritizing parallel methodological streams for pharmaceuticals and devices, while embedding structured stakeholder engagement at each phase. Such clarity will ensure that HTA recommendations are not only technically sound but also implementable across different technology classes. This approach transforms the framework from a conceptual roadmap into a practical tool for governance. In conclusion, the HTA framework may play a vital role in optimizing healthcare delivery in the UAE, enabling the adoption of innovative technologies while maintaining a focus on value for patients.

While the framework offers a structured pathway, its success will depend on factors beyond technical design. Risks include limited availability of high-quality local data, potential delays in legislative adoption, and challenges in sustaining stakeholder engagement across emirates. Furthermore, adapting international best practices to the UAE context may surface unanticipated cultural or systemic barriers. Acknowledging these uncertainties is essential to ensure that HTA implementation remains flexible, iterative, and responsive to evolving national needs.

By sequentially implementing Phase 0 through Phase 3, EHS can ensure that each step, from initial evidence gathering to final policy enforcement, logically builds toward the next. The proposed HTA infrastructure is not just a collection of phases, but a story of progressive capacity building that leads to sustained evidence-based decision-making.
